# A combination of 2 rapid immunoassays significantly improves diagnostic sensitivity for heparin-induced thrombocytopenia

**DOI:** 10.1093/ajcp/aqag016

**Published:** 2026-03-22

**Authors:** Giulia Ciarrocca Taranta, Angela Rogolino, Alessia Bertelli, Andrea Sorrentino, Michela Di Gioia, Francesca Cesari, Betti Giusti, Elena Sticchi, Martina Berteotti, Anna Maria Gori, Rossella Marcucci

**Affiliations:** Department of Experimental and Clinical Medicine, University of Florence, Florence, Italy; Atherothrombotic Diseases, “Careggi” University Hospital, Florence, Italy; Department of Experimental and Clinical Medicine, University of Florence, Florence, Italy; Department of Experimental and Clinical Medicine, University of Florence, Florence, Italy; Atherothrombotic Diseases, “Careggi” University Hospital, Florence, Italy; Atherothrombotic Diseases, “Careggi” University Hospital, Florence, Italy; Department of Experimental and Clinical Medicine, University of Florence, Florence, Italy; Atherothrombotic Diseases, “Careggi” University Hospital, Florence, Italy; Department of Experimental and Clinical Medicine, University of Florence, Florence, Italy; Atherothrombotic Diseases, “Careggi” University Hospital, Florence, Italy; Department of Experimental and Clinical Medicine, University of Florence, Florence, Italy; Atherothrombotic Diseases, “Careggi” University Hospital, Florence, Italy; Department of Experimental and Clinical Medicine, University of Florence, Florence, Italy; Atherothrombotic Diseases, “Careggi” University Hospital, Florence, Italy; Department of Experimental and Clinical Medicine, University of Florence, Florence, Italy; Atherothrombotic Diseases, “Careggi” University Hospital, Florence, Italy

**Keywords:** diagnosis, heparin-induced platelet aggregation test, immunoassay, thrombocytopenia, thrombosis

## Abstract

**Objectives:**

To evaluate the diagnostic performance of chemiluminescent immunoassay (CLIA), latex immunoturbidimetric assay (LIA), and the combination of CLIA/LIA with respect to the functional heparin-induced platelet aggregation (HIPA) test.

**Methods:**

An observational retrospective study was conducted on 100 patients. All samples were initially tested with CLIA on the ACL TOP AcuStar, and then we performed LIA and CLIA tests concurrently (on the same samples on the ACL TOP 970 CL) and the HIPA test.

**Results:**

The CLIA test was performed on both the AcuStar and the ACL TOP 970 CL, and results were concordant: 68% of patients were negative, and 32% were positive. The HIPA test confirmed a diagnosis in 26 of 32 and identified 6 false-positive patients and 1 false-negative patient. The LIA test was performed on the ACL TOP 970 CL: 64% of patients were negative, and the remaining 36% were positive. The HIPA test confirmed a diagnosis in 24 patients and identified 12 false-positive and 3 false-negative patients. The combination of CLIA and LIA tests allowed us to categorize 27 true-positive, 13 false-positive, 0 false-negative, and 60 true-negative patients.

**Conclusions:**

The combination of CLIA/LIA provides high sensitivity with a progressively greater probability of detecting platelet-activating antibodies with a higher assay reactivity, reaching 100% when both automated assays yield moderate or strong results.

## INTRODUCTION

Heparin-induced thrombocytopenia (HIT) is an immune adverse drug reaction induced by exposure to heparin that leads to the production of IgG antibodies against heparin–platelet factor 4 (H-PF4) complexes. Immunocomplex formation results in platelet activation through FcγRIIA receptors, leading to platelet aggregation: HIT is characterized by a significant reduction in platelet count (usually >50%) and an increased risk of arterial and venous thrombosis, typically occurring 5 to 14 days after the initiation of heparin therapy.^1‐4^

Despite progress in understanding the underlying immunologic mechanisms, the diagnosis of HIT remains challenging, as it requires a combination of clinical criteria and specific laboratory tests. However, a rapid diagnosis is essential to prevent potentially catastrophic thrombotic events.^5‐7^

A HIT diagnosis is suspected based on a clinical evaluation with the 4 T pretest probability score (4 T score) and requires the detection of circulating antibodies bound to H-PF4 complexes by immunologic and functional assays.^5^^,^^8‐12^ Immunologic assays are based on different techniques such as chemiluminescent immunoassay (CLIA), latex immunoturbidimetric assay (LIA), and enzyme-linked immunosorbent assay (ELISA). They are easy to perform, widely available, and highly sensitive.^13^ Functional assays are the gold standard for HIT diagnosis after a positive immunologic test. The serotonin release assay (SRA) has traditionally been regarded as the reference test for HIT diagnosis due to its high sensitivity (∼95%) and specificity (∼95%).^8^^,^^14^^,^^15^ The principle of SRA is based on the use of ^14^C-serotonin preincubated with donor platelet-rich plasma.

Because it relies on the use of radioactive ^14^C-serotonin, SRA requires access to laboratories equipped to handle radioactivity and adequately trained laboratory personnel, even though an alternative nonradioactive approach has been developed that measures serotonin release through high-performance liquid chromatography (HPLC).^16‐18^ The comparison of conventional radioactive SRA with HPLC-based SRA showed concordant results.^17^^,^^18^ The limited access to this test, as only a few laboratories are authorized and/or properly equipped to conduct SRA, has led to the adoption of heparin-induced platelet aggregation (HIPA).^19‐21^ All functional tests are technically demanding, time-consuming, and limited to a few expert laboratories.^22^^,^^23^

This study aims to evaluate the performance of CLIA, LIA, and the combination of CLIA/LIA with respect to the functional HIPA test, which serves as the reference for sensitivity and specificity calculations.

## MATERIALS AND METHODS

### Study population

This observational retrospective study investigated 100 patients (42 women, 58 men), for whom clinicians requested H-PF4 antibody detection for suspected HIT. We collected plasma and serum samples that arrived at the Atherothrombotic Diseases Center from April 2020 through September 2024. All stored samples with sufficient material for the required analyses were included in the study. From April 2020 through April 2024, only samples that were both CLIA positive and HIPA positive were stored and analyzed (24/24). From May 2024 through September 2024, both CLIA-positive and CLIA-negative samples were stored, of which 76 of 79 were analyzed. The mean age was 70.5 ±14.6 years, with a range from 22 to 94 years. All patients were admitted to medical wards: 34% from cardiac surgery, 30% from an internal medicine ward, 21% from an intensive care unit, 5% from a COVID-19 ward, 4% from an orthopedic ward, 1% from a neurologic ward, and 5% from other units. Most patients (67%) were on low-molecular-weight heparin (LMWH) treatment, while the remaining 33% were treated with unfractionated heparin (UFH).

The 4 T pretest probability score was assessed by clinicians who requested the laboratory test: 22% of patients had a low-risk probability (0-3), 62% an intermediate-risk probability (4-5), and 16% a high-risk probability (6-8).

**Figure 1:**
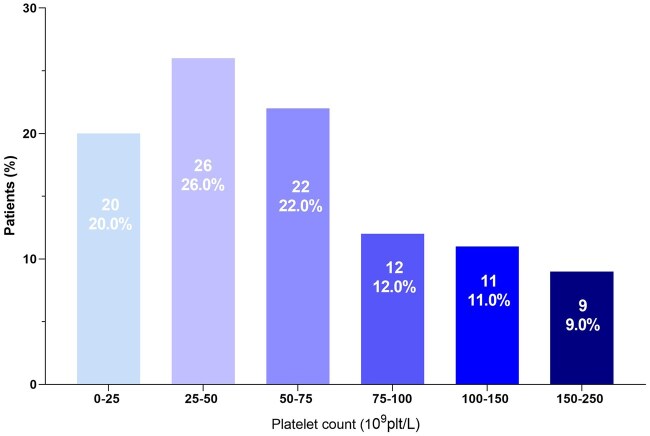
Distribution of thrombocytopenia grades within the study cohort. Platelet count: 0-25 × 10^9^/L: 20% of patients; platelet count: 25-50 × 10^9^/L: 26% of patients; platelet count: 50-75 × 10^9^/L: 22% of patients; platelet count: 75-100 × 10^9^/L: 12% of patients; platelet count: 100-150 × 10^9^/L: 11% of patients; platelet count: 150-250 × 10^9^/L: 9% of patients.

As shown in ** Figure 1,** 86  of 100 (86%) patients experienced a fall in platelet count ≥50%, while the remaining 14% exhibited a reduction <50%. Eighty percent of patients experienced thrombocytopenia, with a nadir platelet count exceeding 25 × 10^9^/L. Severe thrombocytopenia, defined as a nadir platelet count below this threshold, occurred in 20% of the cohort.

### Samples collection and storage

Whole venous blood samples were collected into BD Vacutainer citrated tubes containing 0.109 M trisodium citrate to obtain plasma samples and BD Vacutainer tubes without anticoagulant for serum preparation. Both platelet-poor plasma and serum were obtained by centrifugation at 2500 × *g* for 15 minutes at room temperature and stored in polypropylene tubes at –80 °C until analysis. Samples were stored for up to 4 years and underwent a single thaw at the time of analysis.

All samples were analyzed using CLIA at the time of sample submission according to our laboratory routine. Subsequently, for the purpose of this research project, we performed LIA and CLIA tests simultaneously (on the same sample using the ACL TOP 970 CL; Werfen) and the HIPA test on all 100 samples.

### Immunoturbidimetric assay (HemosIL HIT-Ab)

The new ACL TOP 970 CL was used to analyze plasma samples with a fully automated latex immunoassay for the semiquantitative detection of total immunoglobulins (IgG, IgA, IgM) following the manufacturer’s protocol.

Latex particles are coated with a monoclonal antibody that mimics human HIT antibodies (KKO). The presence of platelet factor 4 (PF4)/polyvinyl sulfonate (PVS) and the patient sample produces a competitive agglutination reaction. KKO on particles binds to PF4/PVS, causing particle agglutination and absorbance increase. The HIT antibodies in plasma sample compete with KKO on particles for PF4/PVS, reducing particle agglutination and absorbance. The instrument calculates the inhibitory antibody concentration by interpolation from a calibration curve, where antibody concentration is inversely proportional to absorbance. For high-value samples (>5.7 U/mL), the ACL TOP 970 CL performs an automated rerun by diluting samples to ensure accurate quantification. A result equal to or greater than 1.00 U/mL is considered a positive result.

### Chemiluminescent assay (HemosIL HIT-IgG)

All plasma samples were routinely analyzed using a fully automated quantitative CLIA for the detection of IgG antibodies against PF4 in complex with heparin. The analysis was performed on the ACL TOP AcuStar (HemosIL AcuStar HIT-IgG PF4-H; Instrumentation Laboratory GmbH) according to the manufacturer’s instructions at the time of sample submission, and samples were stored at –80 °C. Subsequently, frozen aliquots were used to perform the chemiluminescent analysis again, simultaneously with the LIA test, on the ACL TOP 970 CL with the same technology as the ACL TOP AcuStar for the detection of anti–H-PF4 antibodies. In both instruments, a result equal to or greater than 1.0 U/mL is considered a positive result.

Magnetic particles coated with PF4/PVS complex capture, if present, anti–H-PF4 antibodies. A secondary antibody (tracer) labeled with isoluminol causes a luminescent signal, which is directly proportional to the IgG concentration in the sample.

The ACL TOP 970 CL combines a latex immunoturbidimetric assay with the chemiluminescent assay, allowing the 2 methods to be performed simultaneously.

### Functional assay (HIPA)

All serum samples were evaluated using the HIPA assay, as described by Greinacher et al.^21^^,^^24^ Washed platelets from 5 healthy donors were incubated with patient serum in the presence of buffer and therapeutic (0.25 U/mL) and inhibitory (100 U/mL) doses of UFH and LMWH. The test was considered positive if platelet suspensions from at least 2 of 5 different donors were aggregated within 45 minutes, thus confirming the presence of IgG antibodies. The HIPA test was performed as the final step of the diagnostic process, with knowledge of the 4 T score and previous immunoassay results, and the functional test was subsequently repeated for this study. The results of the 2 HIPA tests, performed first according to the diagnostic routine and subsequently for the purposes of the present study, are consistent with 100% agreement.

### Statistical analysis

Data entry and statistical analyses were conducted using SPSS Statistics, version 29.0.2.0 (IBM Corp), and GraphPad Prism 9 software (GraphPad Software).

Passing-Bablok regression analysis was conducted to assess the agreement between the CLIA test on the ACL AcuStar and ACL TOP 970 CL. Bias was evaluated using the Bland-Altman method, which identifies the average of the 2 tests on the x-axis and their difference on the y-axis.

Sensitivity, specificity, positive predictive value (PPV), negative predictive value (NPV), positive likelihood ratio, and negative likelihood ratio were calculated for single LIA and CLIA tests and for their combination. The HIPA test results were used as a reference to identify true-positive and true-negative cases among 100 patients.

Cohen’s κ coefficient was used to assess the agreement between the CLIA test performed on ACL AcuStar and ACL TOP 970 CL, as well as between the HIPA test performed on samples for clinical routine and the HIPA test performed on frozen aliquots for the purpose of the study.

## RESULTS

We report the results according to the different assays.

**Figure 2:**
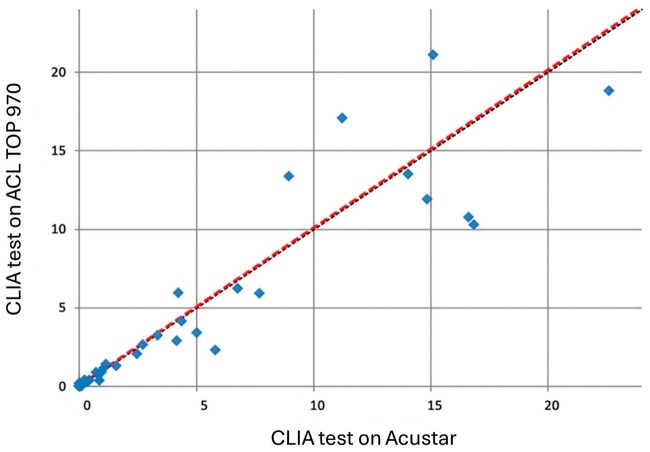
The Passing-Bablok regression analysis between the chemiluminescent immunoassay test on the ACL TOP 970 CL and the ACL AcuStar platforms. Slope: 1.001 [0.94-1.17]; Y-intercept: 0.039. Strong concordance is shown. Two data points (*) reflect very high antibody levels: the AcuStar assay has an upper sensitivity limit of 128 U/mL, whereas the ACL TOP 970 CL platform can provide accurate antibody titers, even in samples exceeding this threshold.

**Figure 3:**
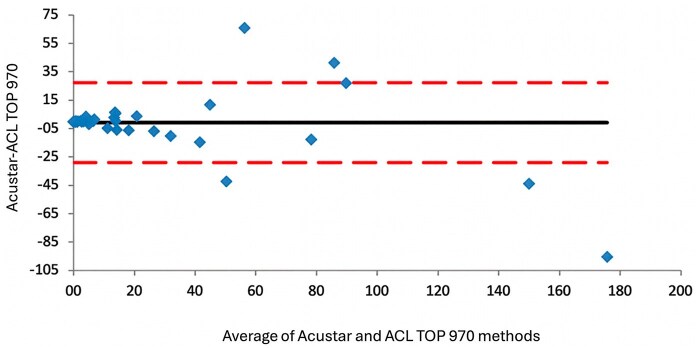
The Bland-Altman plot. The plot shows a strong concordance between the chemiluminescent immunoassay test on the ACL TOP 970 CL and ACL Acustar. Discrepancies appearing in the graph reflect the AcuStar assay’s upper quantification limit of 128 U/mL, whereas the ACL TOP 970 CL platform remains accurate beyond this range.

1. *CLIA test:* Sixty-eight of 100 patients (68%) were negative (<1 U/mL), and 32 (32%) were positive (≥1 U/mL). The CLIA test was performed using both the AcuStar system and the ACL TOP 970 CL, and complete concordance was observed between CLIA results on the ACL AcuStar and ACL TOP 970 CL, with a Cohen’s κ coefficient of 1. Passing-Bablok regression analysis (**Figure 2**; slope: 1.001 [0.94-1.17]; Y intercept: 0.039) and the Bland-Altman plot (**Figure 3**) showed strong concordance between the CLIA test on the ACL TOP 970 CL and the ACL AcuStar. At high antibody levels, we observed some differences, as the AcuStar method has an upper sensitivity limit of 128 U/mL, while ACL TOP 970 CL provides an accurate antibody titer even for high-value samples (>128 U/mL).
2. *LIA test:* Sixty-four of 100 patients (64%) were negative (<1 U/mL), and the remaining 36 (36%) were positive, with a result equal to or greater than 1 U/mL.
3. *HIPA functional test* was performed on all 100 patients and confirmed HIT diagnosis on a total of 27 patients (27%).

According to our diagnostic protocol, all CLIA-positive samples were subsequently analyzed using the functional HIPA test. For the purpose of this study, we began storing the samples that tested negative, and the HIPA test was subsequently performed on all 100 samples. There was a complete concordance between the HIPA results obtained from routinely tested positive samples and the results derived from the subsequent reanalysis, with a Cohen’s κ coefficient of 1.

**Figure 4:**
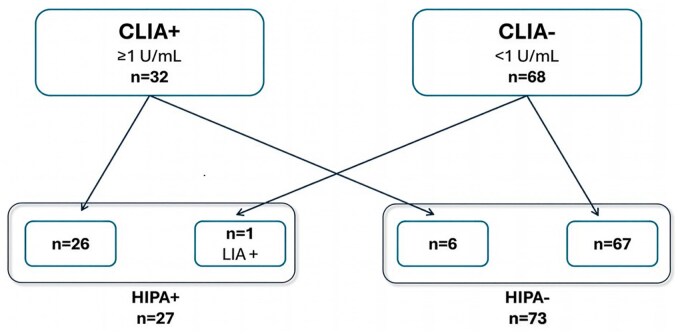
Classification of chemiluminescent immunoassay (CLIA) results according to the heparin-induced platelet aggregation (HIPA) functional assay: among the 32 CLIA-positive patients, 26 were confirmed as positive by the HIPA test, whereas 6 tested negative. Among the 68 CLIA-negative patients, 1 was identified as HIPA positive, while the remaining 67 were confirmed as negative.

**Figure 5:**
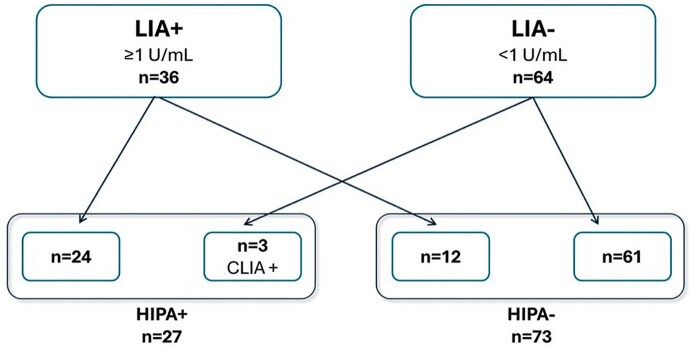
Classification of LIA results according to the heparin-induced platelet aggregation (HIPA) functional assay: among the 36 latex immunoturbidimetric assay (LIA)–positive patients, 24 were confirmed as HIPA positive, whereas 12 tested negative. Among the 64 LIA-negative patients, 3 were identified as HIPA positive, while the remaining 61 were confirmed as negative.

**Figure 6:**
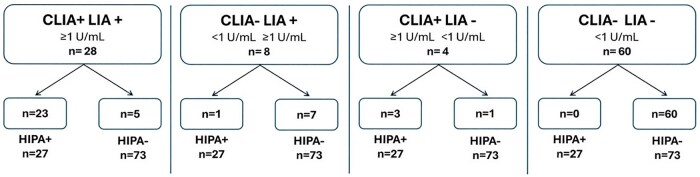
Classification of chemiluminescent immunoassay (CLIA) and latex immunoturbidimetric assay (LIA) combination results according to the heparin-induced platelet aggregation (HIPA) functional assay: among the 28 patients who tested positive on both CLIA and LIA, 23 were confirmed by the functional test, whereas 5 were HIPA negative. Of the 8 patients who tested negative by CLIA but positive by LIA, only 1 was HIPA positive, and the remaining 7 were negative on the functional assay. Four patients tested positive by CLIA but negative by LIA; among these, 3 were HIPA positive, and 1 was HIPA negative. All 60 patients who tested negative on both immunologic assays were confirmed as negative by the functional test.

In detail:

1. Among 32 CLIA-positive patients (≥1 U/mL), the HIPA functional test confirmed HIT diagnosis in 26 patients and excluded HIT in 6 patients. Of 68 CLIA-negative patients (<1 U/mL), the HIPA test resulted positive in only 1 patient and negative in the remaining 67. Based on these results, the CLIA method showed a sensitivity of 96.3% and a specificity of 91.8%. The PPV was 81.25%, and the NPV was 98.53% (**Table 1**). The HIPA test classified 26 true positives, 6 false positives, 1 false negative, and 67 true negatives (**Figure 4**).
2. Concerning LIA, among 36 positive patients, the HIPA test identified 24 positive patients and 12 negative patients; 3 of 64 LIA-negative patients were HIPA positive, while 61 patients were confirmed negative. The LIA test displayed a sensitivity of 88.89% and a specificity of 83.56%. The PPV was 66.67%, and the NPV was 95.31% (**Table 1**). Twenty-four patients were classified as true positives, 12 as false positives, 3 as false negatives, and 61 as true negatives (**Figure 5**).
3. Among 27 HIPA-positive patients, 23 patients were positive on both CLIA and LIA, 1 patient was negative on CLIA and positive on LIA, and 3 patients were positive on CLIA and negative on LIA (**Figure 6**).

**Table 1:** Operating Characteristics of CLIA, LIA, and Dual LIA/CLIA Testing

Characteristic	CLIA test, value (95% CI)	LIA test, value (95%CI)	CLIA + LIA, value (95%CI)
Sensitivity, %	96.3 (81.03-99.91)	88.9 (70.84-97.65)	100 (87.2-100)
Specificity, %	91.8 (82.96-96.92)	83.6 (73.05-91.21)	82.2 (71.5-90.2)
Positive likelihood ratio	11.7 (5.42-25.31)	5.4 (3.17-9.22)	5.6 (3.43-9.19)
Negative likelihood ratio	0.0 (0.01-0.28)	0.1 (0.05-0.39)	0
Positive predictive value, %	81.3 (66.73-90.35)	66.7 (53.97-77.33)	67.5 (55.92-77.27)
Negative predictive value, %	98.5 (90.72-99.78)	95.3 (87.44-98.34)	100 (94.04-100)

Abbreviations: CLIA, chemiluminescent immunoassay; LIA, latex immunoturbidimetric assay.

Of 73 HIPA-negative patients, 60 were negative by both CLIA and LIA, 1 patient was positive for CLIA and negative for LIA, 5 patients were positive on both tests, and 7 patients were negative to CLIA but positive to LIA (**Figure 6**).

By combining CLIA and LIA methods, a sensitivity of 100% and a specificity of 82.2% were achieved. The PPV was 67.5%, and the NPV was 100%. Among the 100 patients, the combination of CLIA and LIA tests allowed us to categorize 27 true positives, 13 false positives, 0 false negatives, and 60 true negative (**Table 1**). Among the 13 false-positive samples identified by combining CLIA and LIA, the 4 T score was 4 in all patients except for 2, whose scores were 1 and 2, respectively. Of these 13 patients, 5 (38.5%) were on UFH, and 8 (61.5%) were on LMWH.

We classified positive samples in 3 categories based on CLIA and LIA results: weak (1.00-3.99 U/mL), moderate (4.00-15.99 U/mL), and strong (≥16 U/mL), assigning a score of 1, 2, or 3 points, respectively, and 0 points for negative samples (<1 U/mL) (**Table 2**). Thus, total points could range from 0 to 6 points. As shown in **Table 3**, as the score increased, a corresponding increase in the percentage of patients who tested HIPA positive was observed. Our analysis further indicated that, among the 60 patients with a CLIA/LIA score of 0, 17 exhibited a low 4 T score (0-3), 36 an intermediate score (4-5), and the remaining 7 a high score (6-8). Notably, all patients with a CLIA/LIA score of 0 tested negative on the HIPA test.

**Table 2:** Sample Classification Based on CLIA and LIA Results

Variable	Points			
	0	1	2	3
CLIA, U/mL	<1.00	1.00-3.99	4.00-15.99	≥16.00
LIA, U/mL	<1.00	1.00-3.99	4.00-15.99	≥16.00

Scored as follows: weak-positive result (1.00-3.99 U/mL) = 1 point, moderate-positive result (4.00-15.9 U/mL) = 2 points, and strong-positive result (≥16.0 U/mL) = 3 points.

Abbreviations: CLIA, chemiluminescent immunoassay; LIA, latex immunoturbidimetric assay.

**Table 3:** Dual CLIA and LIA Tests per the 6-Point Scale (Semiquantitative Analysis)

CLIA/LIA score	HIPA negative, No. (n = 73)	HIPA positive, No. (n = 27)	No. (%) of HIPA-positive patients
0	60	0	0
1	6	2	2/8 (25)
2	5	3	3/8 (37.5)
3	1	1	1/2 (50)
4	1	7	7/8 (87.5)
5	0	8	8/8 (100)
6	0	6	6/6 (100)

Abbreviations: CLIA, chemiluminescent immunoassay; HIPA, heparin-induced platelet aggregation; LIA, latex immunoturbidimetric assay.

## DISCUSSION

Our study demonstrates that combining 2 automated rapid assays—namely, LIA and CLIA—can improve HIT diagnosis. The LIA and CLIA results were compared with those of the HIPA test, one of the reference tests for HIT diagnosis. While SRA is considered the gold standard and widely used in North America, its reliance on radioactive serotonin limits its availability to a small number of adequately equipped laboratories. An alternative to using radioactive material is the measurement of serotonin release via an HPLC-based technique, although it requires advanced instrumentation and trained personnel. Therefore, HIPA is more commonly used in Europe. Only a few studies have directly compared SRA and HIPA in terms of performance characteristics, such as sensitivity and specificity. Two reports have suggested that HIPA may exhibit slightly higher sensitivity compared with SRA in detecting H-PF4 antibodies,^25^^,^^26^ while Greinacher et al^27^ reported similar sensitivity and specificity between the 2 functional tests. As our laboratory is not equipped to perform the SRA assay, we employed the HIPA test as an alternative.

Our results show a strong concordance between the CLIA test performed on the ACL TOP 970 CL and on ACL TOP AcuStar, suggesting that the use of the chemiluminescence assay on the new ACL TOP 970 CL is a reliable tool for HIT diagnosis.

Notably, Marchetti et al^28^ previously observed improved diagnostic performance in HIT by applying the 4 T pretest probability score, combining CLIA as the first-line test, followed by particle-gel immunoassay as a second-line step for patients with a doubtful result, the Lausanne algorithm. Since particle-gel immunoassay production has been discontinued, more recent studies have relied on CLIA- and/or LIA-based approaches.^13^^,^^29^^,^^30^

By using the so-called Hamilton algorithm, Warkentin et al^29^ demonstrated, in a large sample of patients, that the combination of CLIA and LIA enhances the accuracy of HIT diagnosis.

The availability of an instrument (ACL TOP 970 CL) that allows simultaneous testing of CLIA and LIA on the same sample prompted us to conduct this study.

Our results, based on 100 patients with a suspicion of HIT, are consistent with those obtained by Warkentin et al.^29^ Performing both tests in all patients and classifying each patient on the basis of CLIA/LIA reactivities enabled us to accurately predict HIT cases. Interestingly, patients with a score of 0 (n = 60) (both tests <1 U/mL) were all HIPA negative, while patients with a score ≥5 were all HIPA positive (n = 14), suggesting that, in this subset of patients (74% of patients), the functional assay may be unnecessary, and an accurate prediction of HIT can be achieved within an hour of sample arrival in the laboratory by using the CLIA/LIA combination. In the remaining cases (score 1-4, n = 26), the probability of a positive HIPA result increased as the score increased. Only in these cases should we perform the HIPA test to accurately predict HIT. Similarly to the findings by Warkentin et al,^29^ the presence of a CLIA/LIA score ≥4 in our patient population predicted a positive HIPA result in 21 of 22 patients (95%). We also applied the 6-point scoring system proposed by Warkentin et al^29^ to our study population and obtained comparable results; among patients with a 4 T score ≥4, we were able to predict HIT in 20 of 22 cases (90.9%).

The effectiveness of sequential testing—CLIA followed by LIA or vice versa—on diagnostic accuracy for HIT has also been evaluated by different authors. Rittener-Ruff et al^13^ found that using LIA first, followed by CLIA, offered an excellent diagnostic performance in a cohort of 267 patients with suspected HIT. This method detected all HIT cases, with no false positives and a low rate of undetermined results. In contrast, applying the Hamilton algorithm to this cohort^13^ correctly excluded HIT in 85.8% of cases and predicted HIT in 45% of patients, leaving 10.5% of cases unresolved. More recently, Steinauer et al^30^ evaluated over 1000 patients with suspected HIT and confirmed that the approach using LIA as the first test and CLIA as the second demonstrated excellent diagnostic performance, identifying all patients with HIT, with no false HIT predictions, and leaving only 3.4% of unresolved cases. On the other hand, the sequential use of CLIA as the first assay, followed by LIA, resulted in 1 false-positive case and 4.1% of the unresolved cases. Furthermore, the application of the Hamilton algorithm to this cohort of patients^30^ showed reduced predictive power compared to the LIA + CLIA combinations, as only 50% of HIT cases were predicted. Nevertheless, the authors noted that the diagnostic performance of CLIA followed by LIA and LIA followed by CLIA was comparable, correctly predicting HIT in 95.8% and 97.2% of cases, respectively.

In our cohort, the application of sequential algorithms did not improve the identification of HIPA-positive patients. In fact, the approach of using LIA as the first test (≥1 U/mL) would have missed 3 (11%) HIPA-positive samples, 2 of which had an LIA value below the 0.70 U/mL cutoff proposed by Warkentin et al^29^ (0.0 and 0.17 U/mL, respectively). Therefore, according to this cutoff (0.70 U/mL), only 1 case (LIA: 0.81 U/mL) would have proceeded to CLIA testing, but not in the other 2 samples (LIA: 0.0 and 0.17 U/mL). In the LIA-negative sample (0.81 U/mL), the CLIA test resulted positive (≥1 U/mL). In our study, the 3 false-negative patients (LIA-negative and HIPA-positive samples) could not be attributed to the reduced stability of LIA reagents, as suggested by a previous study.^30^ In fact, we performed LIA and CLIA tests on frozen samples, assessing LIA tests in 3 sessions of analysis with internal controls correctly performed every hour and obtaining accurate results.

As concerns the sequential use of CLIA as the first assay (≥1 U/mL), 1 HIPA-positive patient would have been missed, who had a CLIA value of 0.43 U/mL, which is a CLIA value included in the gray zone proposed by Marchetti et al,^28^ but the LIA test was positive (4.15 U/mL).

Applying the gray zone cutoff values from the Lausanne and Hamilton algorithms^28^^,^^29^ to our patient population, a CLIA-first approach would require HIPA testing in 46 patients (CLIA ≥0.13 U/mL), of whom 19 patients had CLIA results between 0.13 and 3.0 U/mL. Therefore, in these patients, we should perform LIA and subsequently HIPA to solve the diagnosis of HIT. According to LIA-first approach, 37 patients had LIA values ≥0.70 U/mL, of whom 24 were in 0.70- to 6-U/mL range, necessitating further testing (HIPA assay).

In line with the recent study by Bissola et al,^31^ our findings support that the combination of CLIA and LIA would be ideal for excluding HIT, as in our sample population, all patients who were negative on both tests were also HIPA negative, with no false-negative patients observed.

We also found that the combined CLIA/LIA approach resulted in a specificity of approximately 82%. Thus, a functional platelet activation assay is recommended to confirm HIT diagnosis, at least in patients with a CLIA/LIA score between 1 and 4.

Based on our data, we envision different roles for the combined CLIA/LIA test approach depending on the resources available in each laboratory.

**Figure 7:**
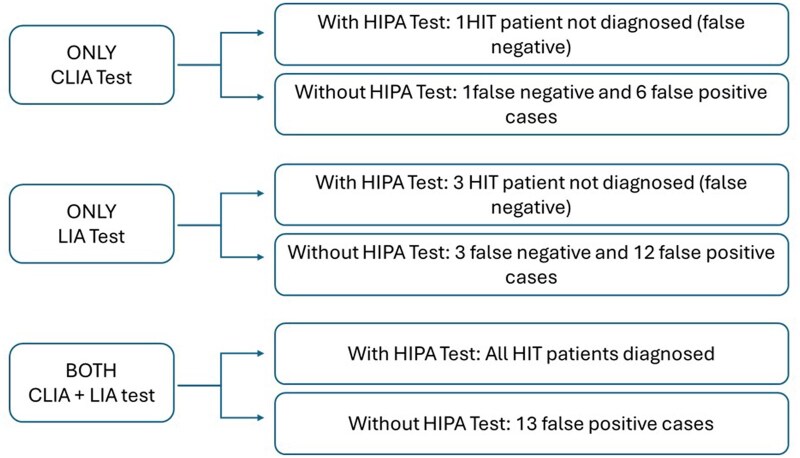
Possible scenarios of a heparin-induced thrombocytopenia diagnosis with chemiluminescent immunoassay, latex immunoturbidimetric assay, and their combination in laboratory settings with and without the availability of the heparin-induced platelet aggregation functional assay.

As illustrated in **Figure 7**, in laboratories with access to functional assays, the combination of CLIA and LIA detects all HIT-positive patients. In laboratories lacking the functional assay, 13 false-positive cases would be diagnosed. Nonetheless, the combination of CLIA and LIA strategy achieves a 100% sensitivity (no patients have been classified as false negative) compared to using only CLIA or LIA tests regardless of HIPA availability (**Figure 7**). Many laboratories in the United States have access to rapid immunoassays such as the LIA and conventional ELISA testing. This combination might provide diagnostic performance comparable to the approach used in the present study, combining LIA and CLIA. However, while CLIA offers automated rapid chemiluminescent detection with high sensitivity, ELISA is more time-consuming and requires manual handling, which can limit its use. Ad hoc studies with the combination of LIA/ELISA tests would be necessary to test this hypothesis.

Our study has limitations: (1) retrospective design, as we have assessed the 2 immunoassays on the new platform in available frozen samples; (2) the limited clinical data; (3) the potential selection bias, since they are not consecutive patients with HIT; (4) the application of specific sample selection criteria, including the exclusion of sample with insufficient volume, which may limit the generalizability of the study findings to other population; and (5) the inability to perform the SRA test, which is considered the gold standard for HIT diagnosis.

The availability of the new platform ACL TOP 970 CL instrument, which allows simultaneous performance of CLIA and LIA tests, reduced equipment costs for 2 instruments. However, testing all samples with both assays incurs additional expenses. In our sample population, the LIA-first approach missed 3 HIPA-positive patients, and the CLIA-first approach missed 1. Thus, the functional HIPA test would still be required for most cases in the LIA-first strategy (as 2 samples had LIA values below 0.70 U/mL), adding significant costs, compared to limiting HIPA testing to 26 patients with a CLIA/LIA score between 1 and 4: in our center, the cost of each immunologic assay (either LIA or CLIA) for the detection of antibodies against H-PF4 complexes is €70.95, whereas the functional assay costs €327. This means that the combination of CLIA and LIA entails a total cost of €141.90, while the addition of the HIPA test results in an overall expense of €468.80 per patient. Therefore, restricting HIPA tests to the 26 patients identified according to our proposed strategy would significantly reduce the costs associated with HIT diagnostic testing.

Future prospective studies are needed to evaluate the real-world performance of these tests and to continuously monitor diagnostic workflows.

These results are extremely useful for clinical practice. Heparin-induced thrombocytopenia is often underdiagnosed, partly due to the absence of the HIPA test in most laboratories. Furthermore, we have demonstrated that even in the rare context in which the HIPA test is available, using a combination of CLIA and LIA tests as immunologic assays improves HIT diagnosis.

## CONCLUSIONS

In conclusion, our study demonstrates that combined CLIA/LIA testing optimizes diagnostic sensitivity, with the probability of detecting platelet-activating antibodies increasing as assay reactivity rises, reaching 100% when both automated assays yield moderate or strong results.

## Data Availability

The data underlying this article will be shared on reasonable request to the corresponding author.
